# Effects of *Lactobacillus plantarum* fermented Shenling Baizhu San on gut microbiota, antioxidant capacity, and intestinal barrier function of yellow-plumed broilers

**DOI:** 10.3389/fvets.2023.1103023

**Published:** 2023-02-22

**Authors:** Weijie Lv, Yimu Ma, Yingwen Zhang, Tianze Wang, Jieyi Huang, Shiqi He, Hongliang Du, Shining Guo

**Affiliations:** ^1^College of Veterinary Medicine, South China Agricultural University, Guangzhou, China; ^2^Guangdong Technology Research Center for Traditional Chinese Veterinary Medicine and Natural Medicine, Guangzhou, China

**Keywords:** Shenling Baizhu San, *Lactobacillus plantarum*, fermentation, growth performance, gut microbiota

## Abstract

The current study focused on the effects of Shenling Baizhu San (SLBZS) fermented by *Lactobacillus plantarum* (*L*. *plantarum*) on gut microbiota, antioxidant capacity, and intestinal barrier function of yellow-plumed broilers. Our results showed that the content of ginsenoside Rb1 was the highest when SLBZS were inoculated with 3% *L*. *plantarum* and fermented at 28°C for 24 h. One-day-old male broilers were divided into five treatment groups. Treatment consisted of a basal diet as a control (Con), 0.1% unfermented SLBZS (U-SLBZS), 0.05% fermented SLBZS (F-SLBZS-L), 0.1% fermented SLBZS (F-SLBZS-M), and 0.2% fermented SLBZS (F-SLBZS-H). On days 14, 28, and 42, six chickens from each group were randomly selected for blood collection and tissue sampling. The results showed that the addition of 0.1% fermented SLBZS could significantly increase average daily feed intake (ADFI) and average daily gain (ADG), and decrease feed conversion ratio (FCR) of broilers. The addition of 0.1 and 0.2% fermented SLBZS significantly increased the lymphoid organ index of broilers on day 28 and 42. The addition of 0.1 and 0.2% fermented SLBZS could improve the antioxidant capacity of broilers. Moreover, the addition of 0.1 and 0.2% fermented SLBZS could significantly increase the villus height/crypt depth of the ileum, and significantly increase the expression of tight junction. In addition, fermentation of SLBZS increase the abundance of *Coprococcus, Bifidobacterium* and *Bilophila* in the gut of broilers. These results indicate that the supplementation of fermented SLBZS in the diet could improve the growth performance, lymphoid organ index, antioxidant capacity, and positively affect the intestinal health of broilers.

## 1. Introduction

As early as 1940s, antibiotics were found to improve the growth efficiency of poultry and swine (1). Since then, an increasing number of reports have corroborated the growth-promoting effects of antibiotics and its application to animal husbandry (1–3). The development of drug resistance in food animals was first reported in 1951 (4). Some studies have shown that the use of antibiotics in food animals produce bacterial resistance and affect human health through the food chain, causing public health problems (5–7). Many countries have reduced or banned the addition of growth promoting antibiotics to animal feed, hence necessity of antibiotics substitute. Studies have shown that many substances have great potential to be substitutes for antibiotics additives, such as plant-derived products with known antibacterial properties derived from herbs and spices, probiotics, prebiotics, antimicrobial peptides and phages (8, 9). Among them, herbal medicine attracts many scientists focusing on application and effectiveness of herbal medicine so as to provide scientific guidance for animal production. In addition to its positive effects and people's historical and traditional experience, herbal medicine has also been proved to be fewer side effects (10) and not easy to cause drug resistance (11), suitable and outstanding to animal feed addition.

Some herbs have been proved to have positive effects on animals when added to feed. The review by Mahmoud Alagawany et al. mentioned that licorice improve the growth performance, immune function and antioxidant capacity of poultry when added to poultry feed (12). Fengjie Ji et al. found that adding *Alpiniae Oxyphyllae* (The Chinese name is Yizhi) to the feed improve the growth performance and maintain intestinal health of Cargill ducks (13). In addition, herbs are deemed to enhance their original properties and/or produce new effects when fermented under appropriate conditions (14). A formula named “Jian Ji San” fermented by *Bacillus subtilis* improved the growth performance of broilers (15). The herbal blend fermented with *Lactobacillus* regulated gut microbiota and immune response, and improve growth performance (16). The fermentation of *sanguismus* by *Lactobacillus plantarum* can effectively inhibit the growth of *Aeromonas hydrophila*, regulate the immune response and improve the survival rate of crucian carp against *Aeromonas hydrophila* (17). Buzhongyiqi decoction, Sijunzi decoction, Shenlingbaizhu decoction and their fermented with *Lactobacillus plantarum* reduce the diarrhea symptoms caused by ceftriaxone sodium and improve intestinal flora and barrier function (18). It can be seen that the application of fermented Chinese herbs in animal husbandry has a broad prospect.

Shenling Baizhu San (SLBZS) is a famous formula in “Tai Ping Hui Min He Ji Ju Fang”. In our previous studies, SLBZS alleviated antibiotic-associated diarrhea by regulating gut microbiota (19), relieved inflammatory bowel disease through multiple pathways (20), and improved DSS-induced colitis by inhibiting the pyroptosis pathway (21). In addition, the theory of Traditional Chinese medicine believes that SLBZS has a good effect on appetizing and gaining weight. This may solve the problem of slow growth of some economic animals. However, there is no report about the application of SLBZS fermentation in broiler breeding.

This study explored the fermentation process of SLBZS and discovered the effect of fermentation SLBZS on growth performance and intestinal barrier function of broilers.

## 2. Materials and methods

### 2.1. Chemical and reagents

Polymyxin B sulfate was purchased from Dalian Meilun Bio-Technology Co., Ltd (Dalian, China). Ginsenoside Rb1 (batch number: PRF20120442) was purchased from Chengdu Prufa Technology Development Co., Ltd (Chengdu, China). De Man Rogosa Sharpe (MRS) agar medium, MRS broth medium and Modified Chalmers (MC) agar medium were all purchased from Qingdao Haibo Biotechnology Co., Ltd (Qingdao, China). Chicken IgA kit and Chicken IgG kit were purchased from Shanghai Mlbio Co., Ltd (Shanghai, China). Glutathione Peroxidase (GSH-PX), Superoxide Dismutase (SOD), Total antioxidant capacity (T-AOC), Malondialdehyde (MDA) kits were purchased from Nanjing Jiancheng Bioengineering Institute (Nanjing, China). Trizol was purchased from Invitrogen (Shanghai, China). DEPC water, ChamQ Universal SYBR qPCR Master Mix, HiScript III RT SuperMix for qPCR, purchased from Vazyme Biotech Co., Ltd (Nanjing, China). The chemicals and reagents used in this study were all pure analytical grade.

### 2.2. Preparation of SLBZS

SLBZS is composed of *Panax Ginseng, Wolfiporia cocos, Atractylodes macrocephala, Dioscorea opposita, Dolichos Lablab, Semen Nelumbinis, Semen Coicis, Fructus Amomi, Platycodon grandiflorus* and *Glycyrrhiza uralensis Fisch*, all herbs were purchased from Beijing Tongrentang Guangzhou pharmaceutical chain Co., Ltd (Guangzhou, China). *Panax Ginseng, Wolfiporia cocos, Atractylodes macrocephala, Dioscorea opposita, Dolichos Lablab, Semen Nelumbinis, Semen Coicis, Fructus Amomi, Platycodon grandiflorus* and *Glycyrrhiza uralensis Fisch* at a ratio of 4:4:4:4:3:2:2:2:2:4, pulverize in a beater and pass through a sieve of 60 mesh (19, 20, 22). 10g SLBZS powder was accurately weighed in a 250 ml conical flask, and 4% (corn flour by weight/substrate by weight) of corn flour was added to it. High-temperature sterilization was performed at 121°C for 20 min, and cooling to room temperature for later use.

### 2.3. Establishment of HPLC method for ginsenoside Rb1

#### 2.3.1. Chromatographic condition

Analysis of constituents in SLBZS was performed on an 1525-2707-2489 series HPLC system (Waters, United States) equipped with a binary solvent manager, sample manager, column compartment, UV detector with 280 nm, and LC-Solution software. The chromatographic column is Sun Fire C18 column (4.6 × 250 mm, 5 μm). Mobile phase A was 0.1% phosphoric acid aqueous solution (1:999, V/V), and mobile phase B was acetonitrile, the gradient elution procedure is shown in Supplementary Table S1. The flow rate was 1 mL /min, the injection volume was 50 μL, the column temperature is 40°C, Uv detection wavelength was selected as 203 nm. HPLC diagram of ginsenoside Rb1 standard was shown in Supplementary Figure S1.

#### 2.3.2. Preparation of reference solution

Accurately weigh 14 mg ginsenoside Rb1 in a 10 mL volumetric flask and add methanol to 10 mL to prepare a reference solution containing ginsenoside Rb1 1400 μg per 1 mL methanol. Seal the solution with a sealing film and store at 4°C for later use.

#### 2.3.3. Preparation of the test solution

Accurately weigh 5 g powder to be tested and placed in a 50 mL conical flask, 10 mL of 70% methanol was added, the plug was covered, its quality was recorded, and the water temperature was controlled at 40°C. The ultrasonic was 45 min and stirred every 15 min with ultrasonic power of 50 kHz. Let it sit overnight, ultrasound again for 45 min, cool it at room temperature, weigh the weight again, use 70% methanol to make up the weight lost, mix well, transfer it to 15 mL centrifuge tube, centrifuged at 4,000 rpm for 10 min, the supernatant was filtered by 0.22 μm microporous membrane to obtain the test solution.

### 2.4. Bacterial cultures and growth conditions

*Lactobacillus plantarum* (*L. plantarum*) was grown in MRS broth medium at 37°C without shaking. After 24 h, the final concentration of the bacterial solution was 2 × 10^9^ CFU/mL, which was inoculated into the new MRS Broth medium at the rate of 2% for extended culture. After 24 h of culture in a shaker, centrifuged at 4000 rpm for 10 min, take the bacterial mud for use.

### 2.5. Exploration of fermentation conditions

SLBZS was fermented by *L. plantarum*, and the content of ginsenoside Rb1 in Shenlingbaizhu Powder after fermentation was used as the index. The bacterial inoculum, fermentation temperature and fermentation time were explored. Single factor levels are shown in Supplementary Table S2.

#### 2.5.1. Effect of bacterial inoculum on fermentation

Add SLBZS to 5 mL of distilled water, the ammonium sulfate content (as a source of N) is 0.2%, the fermentation temperature was 28°C, the fermentation time was 36 h, the bacterial inoculum was 1, 3, 5, 7, 9% (V/W bacterial mud volume/ substrate by weight), respectively. The experiment was repeated three times and the results were averaged. The content of ginsenoside Rb1 was obtained.

#### 2.5.2. Effect of fermentation temperature on fermentation

Add SLBZS to 5 mL of distilled water, the ammonium sulfate content is 0.2%, the bacterial inoculum was 3% (V/W bacterial mud volume/substrate by weight), the fermentation time was 36 h, and the fermentation temperature was 24, 28, 32, 36, 40°C, respectively. The experiment was repeated three times and the results were averaged. The content of ginsenoside Rb1 was obtained.

#### 2.5.3. Effect of fermentation time on fermentation

Add SLBZS to 5 mL of distilled water, the ammonium sulfate content is 0.2%, the bacterial inoculum was 3% (V/W bacterial mud volume/ substrate by weight), the fermentation temperature was 28°C, and the fermentation time was 12, 24, 36, 48, 72 h, respectively. The experiment was repeated three times and the results were averaged. The content of ginsenoside Rb1 was obtained.

#### 2.5.4. Calculation of ginsenoside Rb1 yield

Calculation method of ginsenoside Rb1 yield:


$$\begin{array}{l} \text{Ginsenoside Rb1 yield}=\frac{V\;\times \;\rho n}{1000\;\times \;67\%\;\times \;M} \end{array}$$

V: volume of test solution, in mL. ρn: mass concentration of ginsenoside Rb1, in μg/mL. M: mass of powder to be tested, in g. The unit of yield of ginsenoside Rb1 was mg/g.

### 2.6. Determination of pH value

Accurately weigh 2.0 g of powder to be measured in a beaker, add 1 mL distilled water, stir evenly, add 20 mL distilled water, stir for 5 min, then stand for 20 min, determine the pH value of supernatant with a precision pH meter.

### 2.7. Determination of *L. plantarum* concentration

The powder to be measured was precisely weighed 2.0 g in a 50 mL conical flask, and 18 mL sterilized normal saline was added. The powder was stirred and mixed with a glass rod, and was incubated at 37°C at 140 r/min and shaken for 45 min to obtain a dilution of 1:10. In the clean bench, dilute tenfold, successively to 10^−9^. Choose 3–4 suitable gradients, absorb 1 mL of diluent from each gradient into a sterile plate, Add 15 mL of modified MC medium (10,000 IU polymyxin B sulfate per 100 mL MC agar) in time. Shake the dish to mix thoroughly, and do two repetitions for each dilution. At the same time, 1 mL sterilized normal saline for dilution was used instead of diluent as blank control. Let stand for 40–50 min, turn the plate upside down, culture at 37°C for 36–48 h, and select colony count by transparent ring. Calculation formula: *L. plantarum* concentratio*n* = average colony × dilution ratio.

### 2.8. Animals and experimental design

One hundred and sixty healthy 1-day-old male yellow-feathered broilers were purchased from Guangdong Wens Dahuanong Biotechnology Co. Ltd (Guangdong, China). All experimental procedures used in this study were approved by the Animal Ethics Committee of the South China Agricultural University (approval number: SYXK 2019-0136, Guangzhou, China). The care and use of all animals were performed according to the Guidelines for Animal Experiments of the South China Agricultural University.

Broilers were randomly assigned into 5 treatment groups with 4 replicates (cages) per treatment and 8 birds per replicate. Treatments were as followed (Table 1): (1) control (Con) group, (2) unfermented SLBZS (U-SLBZS) group, (3) fermentation SLBZS low dose (F-SLBZS-L) group, (4) fermentation SLBZS medium dose (F-SLBZS-M) group, (5) fermentation SLBZS high dose (F-SLBZS-H) group. The experiment lasted for 42 days, with 1–14 days as the first stage, 15–28 days as the second stage, and 29–42 days as the third stage. Broilers were raised in cages without immunization and were free to eat and drink during the experiment, feed composition and nutrient level are shown in Supplementary Table S3. In the first week, the lighting time was controlled at 23 h, the darkness time was 1 h, and the temperature was controlled at 33–35°C. From the second week, the lighting time was controlled at 18 h, the darkness time was 6 h, and the temperature was gradually reduced from 33–35 to 25°C. Feed intake of broilers was recorded every day. On days 14, 28 and 42, 6 broilers were randomly selected from each group and fasted for 12 h. Body weight was recorded and blood was collected from jugular vein. The collected blood was centrifuged at 3000 rpm for 10 min, and serum was stored in −80°C for testing. After the broiler was euthanized, its viscera and cecal contents were collected and its weight was recorded, the viscera and cecal contents were stored in −80°C. Organ index = organ weight/body weight × 100%.

**Table 1:** Grouping of broilers.

**Group**	**Treatment**
Control group (Con)	None
Unfermented SLBZS group (U-SLBZS)	Add 0.1% unfermented SLBZS
Fermentation SLBZS low dos group (F-SLBZS-L)	Add 0.05% fermented SLBZS
Fermentation SLBZS medium dose group (F-SLBZS-M)	Add 0.1% fermented SLBZS
Fermentation SLBZS high dose group (F-SLBZS-H)	Add 0.2% fermented SLBZS

### 2.9. ELISA

ELISA kit was used to quantify the contents of IgA and IgG in serum of broilers. The absorbance at 450 nm was measured at 37°C on a Mltiskan FC Automatic microplate reader (Thermo Fisher Scientific, Massachusetts, USA) to determine factor content levels.

### 2.10. Histological procedures

Tissue was collected and fixed in 10% buffered formalin, embedded in paraffin and sliced into 5-μm-thick sections. Tissues were stained with H&E, and slides were assessed for villi and crypt structure.

### 2.11. Tissue RNA extraction and qRT-PCR analysis

Total RNA from the liver tissue was extracted using Trizol (Vazyme Biotech Co.,Ltd, Nanjing, China),and a Takara PrimeScript RT Kit (Vazyme Biotech Co., Ltd, Nanjing, China) was used to transcribe RNA into cDNA. ChamQ Universal SYBR qPCR Master Mix (Vazyme Biotech Co., Ltd, Nanjing, China) was used for qRT-PCR following the manufacturer's instructions. NCBI Primer-BLAST (USA) was used to design the primers. The primer sequences are shown in the Supplementary Table S5.

### 2.12. 16S rRNA sequencing analysis

DNA extraction from broilers fecal samples was performed using DNeasy PowerSoil Kit (MoBio/QIAGEN) according to the manufacturer's instructions. The absorbance values of DNA at 260/280 nm were measured using a fluorescence spectrophotometer to assess the concentration of sample DNA. The quality of DNA was detected by 1% agarose gel electrophoresis. The V3-V4 region of the microbial 16S rRNA gene was amplified by PCR. The primer sequences were F: ACTCCTACGGGAGGCAGCA, R: GGACTACHVGGGTWTCTAAT. Sequencing was performed by Shanghai Paisano Biotechnology Co., Ltd. (Shanghai, China) using Illumina MiSeq gene sequencing platform.

### 2.13. Statistical analysis

The data were analyzed using SPSS 22.0 software (IBM, Armonk, NY, USA). GraphPad Prism 7.0 software (GraphPad; San Diego, CA, USA) was used to generate graphs. All data are presented as the mean value ± SD of at least three independent experiments. The data were ANOVA, Least-SignificantDifference (LSD) and Duncan's. *p* < 0.05 was considered significant.

## 3. Results

### 3.1. *L. plantarum* fermentation improve the content of ginsenoside Rb1 in SLBZS

Probiotic fermented herbs have been proved to improve the effect. For example, Danggui Buxue Tang fermented by *L. plantarum* enhanced its anti-diabetes function (23), Cherries fermented with *L. plantarum* improved immunity in immunosuppressed mice (24). Therefore, we explored the content changes of SLBZS before and after *L. plantarum* fermentation. Firstly, the effects of bacterial inoculation amount, fermentation temperature and fermentation time were investigated by single factor test. Single factor levels are shown in Supplementary Table S2. Ginsenoside Rb1 is one of the main components of SLBZS, and the content of ginsenoside Rb1 was taken as the index. Through single factor test, 5% bacterial inoculation, 28°C fermentation temperature and 36 h fermentation time were selected as the fermentation conditions (Supplementary Figure S2). In order to continue to optimize fermentation conditions, we designed an orthogonal experiment (Table 2), and the levels of each factor in the orthogonal test are shown in Supplementary Table S4. According to the *K*-value in the orthogonal experiment (Table 2), the best combination of fermentation conditions was 3% of bacterial inoculation, 28°C and fermentation for 24 h. Therefore, 3% of bacterial inoculation, fermentation temperature at 28°C and fermentation time at 24 h were selected for subsequent tests.

**Table 2:** Orthogonal experimental design and results.

**Test group**	**A: Bacterial inoculation amount**	**B: Fermentation temperature**	**C: Fermentation time**	**Ginsenoside Rb1 yield (mg/g)**
1	1	1	1	15.96
2	2	2	2	20.08
3	3	3	3	18.83
4	1	2	3	20.38
5	2	3	1	20.37
6	3	1	2	14.52
7	1	3	2	19.70
8	2	1	3	13.78
9	3	2	1	19.86
K1	56.04	44.26	56.19	
K2	54.23	60.33	54.30	
K3	53.21	58.89	52.99	
k1	18.68	14.75	18.73	
k2	18.08	20.11	18.10	
k3	17.74	19.63	17.66	
R	0.94	5.36	1.07	

K means the sum of data at a certain level in the column. k means the average of the sum of data at a certain level in this column. R means the range of k in this column.

**Figure 1:**
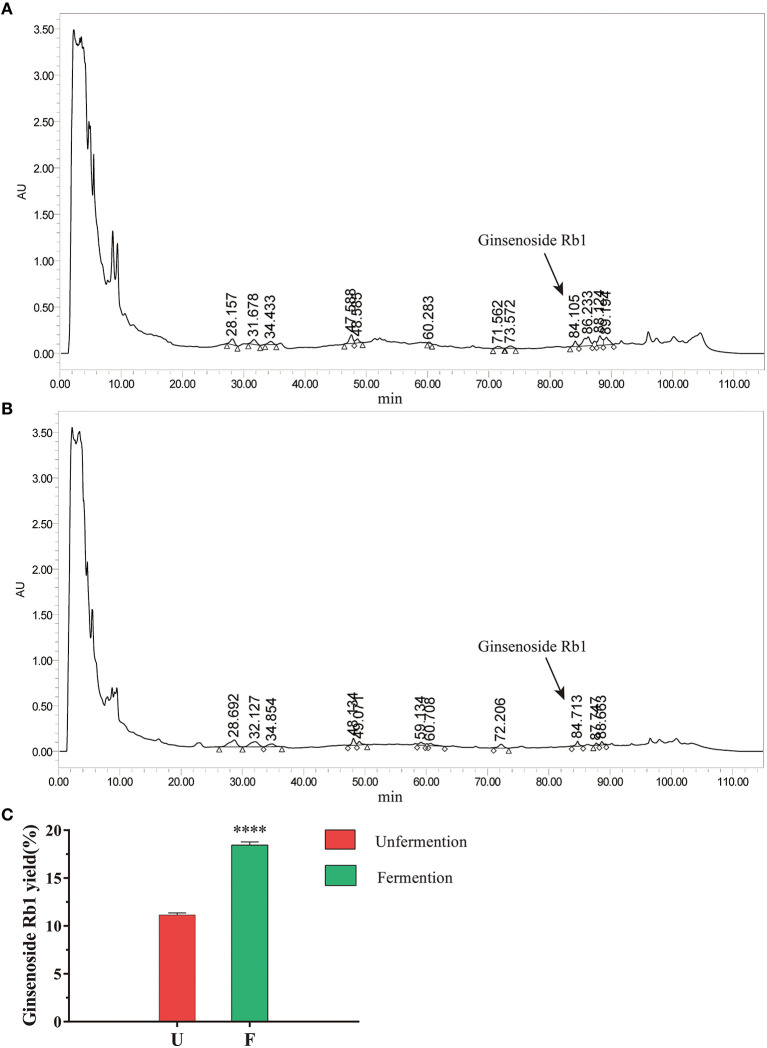
Effects of *L. plantarum* fermentation on SLBZS. **(A)** Chromatograms of unfermented SLBZS. **(B)** Chromatogram of fermented SLBZS. **(C)** The yield of ginsenoside Rb1 before and after SLBZS fermentation. Bars represent mean values ± SD (*n* = 3). ^****^ indicate *p* < 0.0001 compared with the Unfermention.

Next, we used the above fermentation conditions to ferment SLBZS, and ginsenoside Rb1 was analyzed by HPLC. According to the chromatogram of ginsenoside Rb1 standard, the peak of ginsenoside Rb1 was around 84.261 min (Supplementary Figure S1). When compared the chromatograms of SLBZS before and after fermentation the composition and content of SLBZS changed after fermentation (Figures 1A, B). The results showed that compared with unfermentation, the content of ginsenoside Rb1 in SLBZS after *L. plantarum* fermentation was significantly increased (Figure 1C). In addition, the enrichment of *L. plantarum* was associated with decreased pH after fermentation (Supplementary Figure S3). In summary, these results suggested that *L. plantarum* fermentation improve the content of ginsenoside Rb1 in SLBZS and may enhance the effect of SLBZS.

### 3.2. Fermentation of SLBZS improve the growth performance of broilers

**Figure 2:**
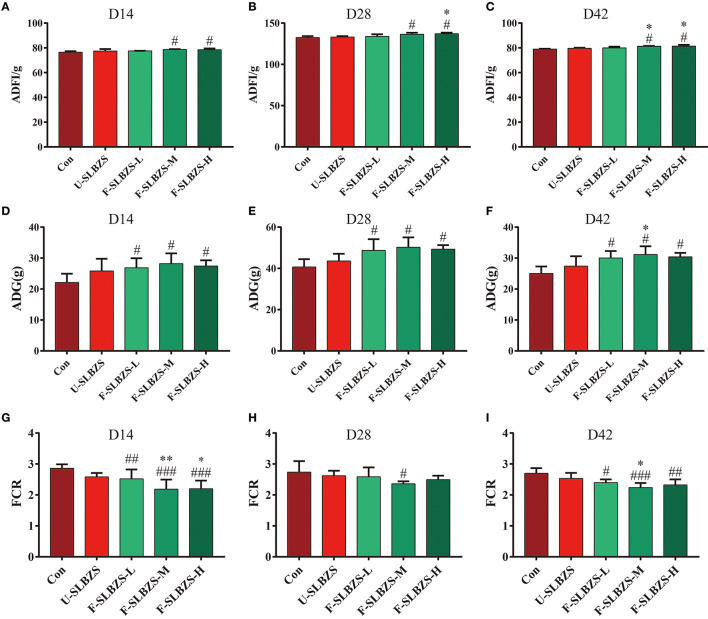
Effects of fermented SLBZS on growth performance of broilers. **(A)** ADFI of broilers in the second stage. **(B)** ADFI of broilers in the third stage. **(C)** ADFI of broilers in the whole stage. **(D)** ADG of broilers in the second stage. **(E)** ADG of broilers in the third stage. **(F)** ADG of broilers in the whole stage. **(G)** FCR of broilers in the second stage. **(H)** FCR of broilers in the third stage. **(I)** FCR of broilers in the whole stage. Bars represent mean values ± SD (*n* = 3 for ADFI, *n* = 4–6 for ADG and FCR). ^#^, ^##^, and ^###^ indicate *p* < 0.05, *p* < 0.01 and *p* < 0.001 compared with the Con group, ^*^ and ^**^ indicate *p* < 0.05 and *p* < 0.01 compared with the U-SLBZS group.

Some probiotic-fermented herbal promoted the growth of chicks (16) or broilers (25). In our experiment, ADFI, ADG and FCR of broilers were measured at days 1–14 (the first stage), 15–28 (the second stage), 29–42 (the third stage) and 1–42 (whole stage). The results showed that the supplementation of either unfermented SLBZS or fermented SLBZS had no significant effect on growth performance in the first stage (Supplementary Figure S4). In the second stage, medium dose and high dose of fermented SLBZS improved the ADFI (Figure 2A), and three doses of fermented SLBZS significantly improved the ADG (Figure 2D) and reduced FCR of broilers (Figure 2G). In the third stage, medium dose and high dose of fermented SLBZS improved the ADFI of broilers (Figure 2B), three doses of fermented SLBZS significantly improved the ADG of broilers (Figure 2E), only medium dose of fermented SLBZS significantly reduced the FCR of broilers (Figure 2H). For the whole stage, medium dose and high dose of fermented SLBZS significantly increased the ADFI of broilers (Figure 2C), and three doses of fermented SLBZS increased the ADG (Figure 2F) and reduced the FCR of broilers (Figure 2I). The results suggested that adding unfermented or fermented SLBZS into the feed improved the growth performance of broilers, and the effect of fermented SLBZS is more effective.

### 3.3. Fermentation of SLBZS increased the lymphoid organ index and immune factors of broilers

**Figure 3:**
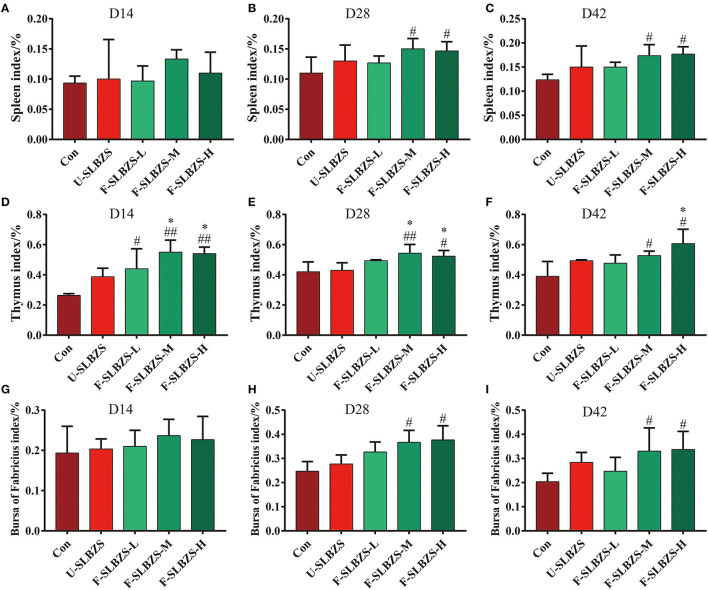
Effects of fermented SLBZS on lymphoid organs weight of broilers. **(A)** Spleen index of broilers on day 14. **(B)** Spleen index of broilers on day 28. **(C)** Spleen index of broilers on day 42. **(D)** Thymus index of broilers on day 14. **(E)** Thymus index of broilers on day 28. **(F)** Thymus index of broilers on day 42. **(G)** Bursa of Fabricius index of broilers in on day 14. **(H)** Bursa of Fabricius index of broilers on day 28. **(I)** Bursa of Fabricius index of broilers on day 42. Bars represent mean values ± SD (*n* = 3). ^#^ and ^##^ indicate *p* < 0.05 and *p* < 0.01 compared with the Con group, ^*^ indicate *p* < 0.05 compared with the U-SLBZS group.

Next, we tested whether adding fermented SLBZS to the feed had an effect on the immune function of broilers. The results showed the addition of medium and high doses of fermented SLBZS significantly increased the spleen index, thymus index and bursa of fabricius index of broilers on day 28 and 42 (Figures 3B, C, E, F, H, I). In addition, three doses of fermented SLBZS on day 14 significantly increased thymus index of broilers (Figure 3D), but had no significant effect on spleen index and bursa of fabricius index (Figures 3A, G). We wondered whether adding fermented SLBZS to the feed had an effect on the expression of antibody. To verify the conjecture, we tested the content of IgA and IgG in serum. On day 14, serum IgA in low dose and high dose of fermented SLBZS was significantly higher than that of normal diet (Supplementary Figure S5A), however, IgG content did not increase significantly (Supplementary Figure S5D). On day 28 and day 42, serum IgA content showed an increasing trend but was not significant (Supplementary Figures S5B, C). Serum IgG content was significantly increased on day 28 when adding low dose and medium dose of fermented SLBZS, and significantly increased on day 42 when adding unfermented SLBZS and high dose of fermented SLBZS (Supplementary Figures S5E, F). In general, the result suggested that adding fermented SLBZS can increase the lymphoid organ index and immune factors of broilers.

### 3.4. Fermentation of SLBZS enhance the antioxidant capacity of broilers

**Figure 4:**
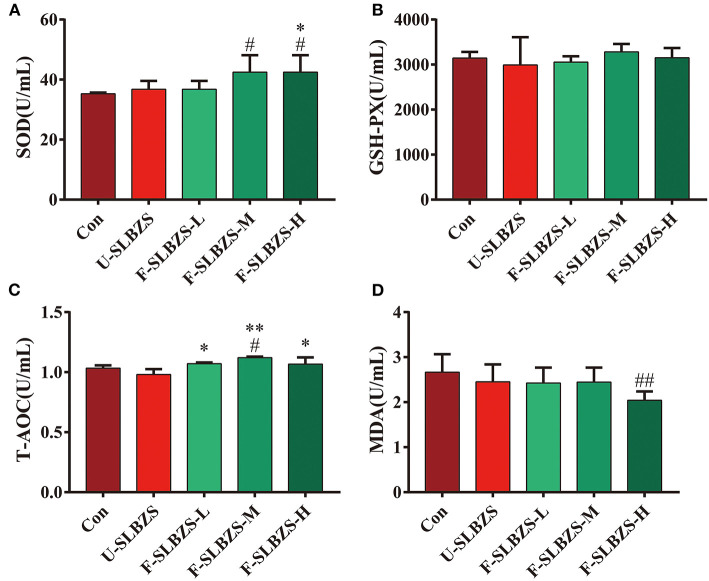
Effects of fermented SLBZS on antioxidant factor content in broilers. **(A)** Content of SOD in serum of broilers. **(B)** Content of GSH-PX in serum of broilers. **(C)** Content of T=AOC in serum of broilers. **(D)** Content of MDA in serum of broilers. Bars represent mean values ± SD (*n* = 3-5). ^#^ and ^##^ indicate *p* < 0.05 and *p* < 0.01 compared with the Con group, ^*^ and ^**^ indicate *p* < 0.05 and *p* < 0.01 compared with the U-SLBZS group.

Researchers found that adding herbal ingredients or fermented preparations to broiler feed increased antioxidant capacity (26–28). In order to determine whether adding fermented SLBZS to the feed affect the antioxidant capacity of broilers, we tested the serum antioxidant factors of broilers according to the operating instructions. The current study exhibited a significantly increase of SOD level in broilers when adding medium and high dose of fermented SLBZS (Figure 4A), but an unapparent increasing trend of GSH-PX (Figure 4B). Adding medium dose of fermented SLBZS significantly increased T-AOC level of broilers (Figure 4C), and high dose of that significantly reduced MDA content (Figure 4D). Moreover, compared with unfermented SLBZS, adding fermented SLBZS significantly increased the T-AOC of broilers (Figure 4C). Therefore, our results indicated that adding fermented SLBZS to broiler feed can improve antioxidant capacity of broiler.

### 3.5. Fermentation of SLBZS improve ileum villus morphology of broilers

**Figure 5:**
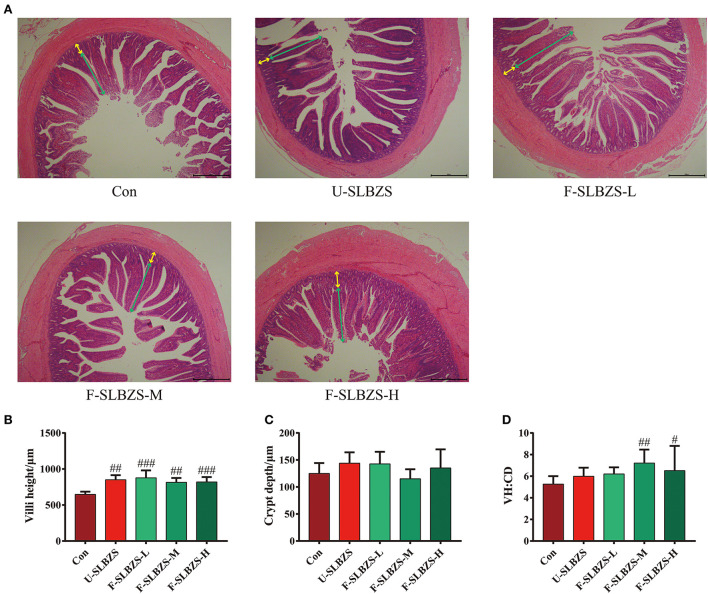
Effects of fermented SLBZS on intestinal morphology of broilers. **(A)** H&E of broiler ileum. Green arrow: villi height, Yellow arrow: crypt depth. Scale: 200 μm. **(B)** Villi height in ileum of broilers. **(C)** Crypt depth of ileum in broilers. **(D)** Villi height to crypt depth (VH:CD) ratio ileum of broilers. Bars represent mean values ± SD (*n* = 5). ^#^ and ^##^ indicate *p* < 0.05 and *p* < 0.01 compared with the Con group.

To better understand differences between broilers added fermented SLBZS and unfermented SLBZS, we investigated the variety of ileum villus morphology in two groups since it plays a crucial role in nutrients absorption. The result showed that both unfermented SLBZS and fermented SLBZS could significantly increase ileum villi height (VH) of broilers (Figures 5A, B). There was no significant change in crypt depth (CD) (Figure 5C). In addition, medium and high dose of fermented SLBZS significantly increased the villi height to crypt depth (VH:CD) ratio in ileum of broilers (Figure 5D). The increase in VH:CD ratio indicated increase in the intestinal surface area for nutrient absorption, which may contribute to improved growth performance (29, 30). The results suggested that adding fermented SLBZS can improve the broiler initiation morphology.

### 3.6. Fermentation of SLBZS enhance the intestinal barrier function of broiler cecum

**Figure 6:**
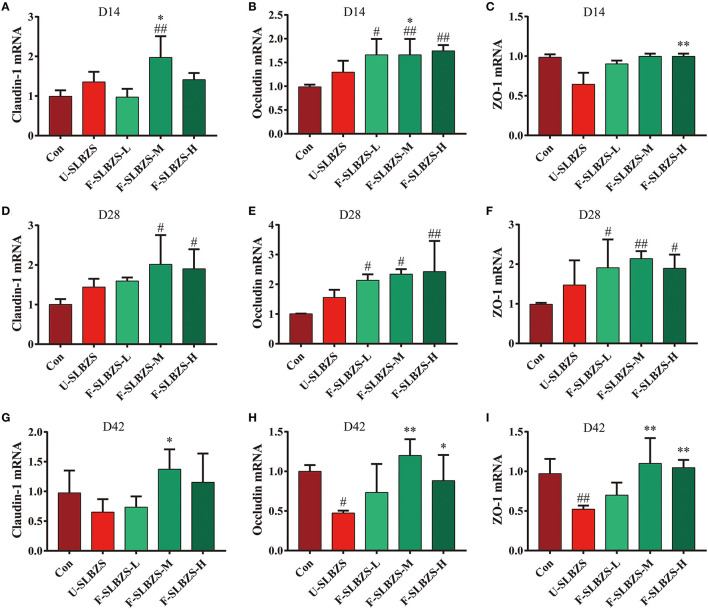
Effects of fermented SLBZS on ileum intestinal barrier in broilers. **(A)** Relative mRNA expression of Claudin-1 in broiler cecum on day 14. **(B)** Relative mRNA expression of Occludin in broiler cecum on day 14. **(C)** Relative mRNA expression of ZO-1 in broiler cecum on day 14. **(D)** Relative mRNA expression of Claudin-1 in broiler cecum on day 28. **(E)** Relative mRNA expression of Occludin in broiler cecum on day 28. **(F)** Relative mRNA expression of ZO-1 in broiler cecum on day 28. **(G)** Relative mRNA expression of Claudin-1 in broiler cecum on day 42. **(H)** Relative mRNA expression of Occludin in broiler cecum on day 42. **(I)** Relative mRNA expression of ZO-1 in broiler cecum on day 42. Bars represent mean values ± SD (*n* = 3). ^#^ and ^##^ indicate *p* < 0.05 and *p* < 0.01 compared with the Con group, ^*^ and ^**^ indicate *p* < 0.05 and *p* < 0.01 compared with the U-SLBZS group.

Based on the result that adding fermented SLBZS can improve the broiler intestinal morphology, we explored the effects of fermented SLBZS on the intestinal barrier by detecting the RNA relative expression of Claudin-1, Occludin and Zonula Occludens-1 (ZO-1). We noticed, on day 14, that medium dose of fermented SLBZS increased the mRNA expression of Claudin-1, while low, medium and high dose of fermented SLBZS increased the mRNA expression level of Occludin (Figures 6A, B). All three doses had no significant effect on the mRNA expression level of ZO-1 (Figure 6C). On day 28, both medium and high doses of fermented SLBZS increased the mRNA expression of Claudin-1, while all three doses of fermented SLBZS increased the mRNA expression of Occludin and ZO-1 (Figures 6D–F). On day 42, medium dose fermentation SLBZS increased the mRNA expression of Occludin, and medium dose and high dose fermentation SLBZS increased the mRNA expression of Claudin-1 and ZO-1 (Figures 6G–I). However, on day 42, the addition of unfermented SLBZS and low dose fermented SLBZS reduced the expression of Claudin-1, Occludin, and ZO-1 (Figures 6G–I). In conclusion, adding medium dose of fermented SLBZS to broiler feed can increase the mRNA expression of tight junction protein in cecum of broilers. The result suggested that fermented SLBZS can enhance the cecal barrier function of broilers.

### 3.7. Fermentation of SLBZS altered the gut microbiota of broilers

**Figure 7:**
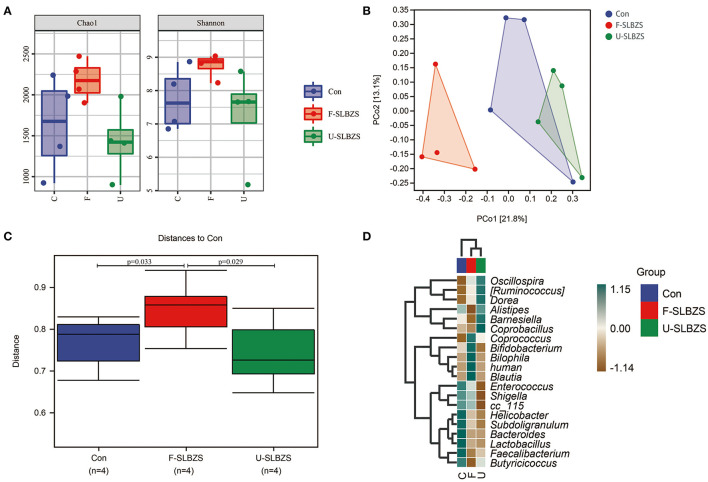
Fermented alters the structure of the gut microbiota in broilers. **(A)** Alpha analysis, including Chao1 and Shannon. **(B)** Principal Co-ordinates Analysis (PCoA). **(C)** Between-groups difference analysis. **(D)** Heatmap of species composition.

In view of the effect of fermented SLBZS on the gut, we examined the gut microbiota of broilers. Compared with Con and U-SLBZS, Chao1 and Shannon index in F-SLBZS group were increased, indicating that the microbiota species increased, and the abundance and evenness increased (Figure 7A). Unifrac-based PCoA analysis demonstrated that F-SLBZS was clustered separately compared with Con and U-SLBZS (Figure 7B). These results indicated that the addition of fermented SLBZS significantly change the gut microbiota structure of broilers, while the addition of SLBZS had a limited effect on it. According to between-groups difference analysis, the gut microbiota of broilers fed fermented SLBZS changed significantly (Figure 7C). The heatmap of species composition showed that the abundance of *Coprococcus, Bifidobacterium, Bilophila, human* and *Blautia* in F-SLBZS were higher than those in Con and U-SLBZS (Figure 7D).

## 4. Discussion

Antibiotic growth promoters could improve the growth performance of livestock and poultry, and have obvious benefits on the structure and function of intestinal epithelium, which has been considered as the “gold standard” to improve the performance of feed additives, and provided a direction for finding substitutes (31). However, with the wide spread of multi-drug resistant bacteria, public health has been seriously threatened. Many countries have banned antibiotics in feed, hence the need for its substitutes. Over the years, there are growing studies propose phytogenic products the most likely substitutes for antibiotics added to livestock and poultry feed (8, 9). For example, Pirgozliev et al. showed the growth performance, energy and nutrient retention and the intestinal cytokine expression of broilers could be improved by adding 5% carvacrol, 3% cinnamaldehyde and 2% capenne oleoresin to their diets (32). Besides, Galli et al. found that adding additives containing thymol, cinnamaldehyde and carvacrol to broiler feed could improve growth performance and meat quality without compromising intestinal health (33). Gholami-Ahangaran et al. suggested that the addition of Gunnera (*Gundelia tournefortii L*.) extract and protein to broiler feed had a synergistic effect on feed efficiency and antioxidant status and reduced lipid levels, while having no effect on liver function of broilers (34). Our previous studies showed that SLBZS could alleviate antibiotic diarrhea (19) and colitis induced by DSS (20–22). Keeping healthy of the gut facilitates the absorbtion of nutrition (35, 36). Therefore, we proposed that adding SLBZS into broiler feed as a feed additive would promote the growth performance of broilers.

Besides, in the process of studying phytogenic products, the very promising fermented herbal medicines drew our attention for their enhancements to their original properties and/or generations of new effects (14). Gao et al. found that the addition of Chinese medicine–probiotic compound microecological preparation, which is composed of 5 traditional Chinese medicine herbs (*Galla Chinensis, Andrographis paniculata, Arctii Fructus, Glycyrrhizae Radix*, and *Schizonepeta tenuifolia*) fermented by *Aspergillus niger* and a kind of compound probiotics (*L. plantarum A37* and *L. plantarum MIII*), could improve the growth performance, serum parameters, immune function and intestinal health of broilers (25). Wang et al. found that adding fermented herbal preparations, which is composed of 4 traditional Chinese medicine herbs (*Astragalus, P.notoginseng*, licorice and chickpeas) fermented by *L. paracasei* KL1 and *L. plantarum* Zhang-LL, can improve growth performance, regulate gut microbiota and enhance immunity of broilers (16).

Studies have reported that different probiotics were selected for fermentation, such as *L. plantarum, Lactobacillus paracei* and *Bacillus* (16, 18, 23, 37). Based on these previous studies, we selected *L. plantarum* for fermentation and explored the best fermentation conditions. Ginsenoside Rb1 is often considered as one of the active components of SLBZS, so we chose ginsenoside Rb1 as the fermentation index. Bacterial inoculum on fermentation, fermentation temperature and fermentation time are often the variables that need to be controlled (15, 38). Orthogonal experiments are often used to analyze the optimal combination of multifactor levels and to screen fermentation conditions (25). Through orthogonal experiments, 3% of bacterial inoculum, 28°C and 24 h of fermentation were selected as the conditions. To determine the occurrence of fermentation, we tested *L. plantarum* content and pH before and after fermentation. *Lactobacillus* fermented carbohydrate to lactic acid or other acids, and decrease the pH (39, 40). The results showed the content of *L. plantarum* increased significantly and that the pH decreased significantly after fermentation, indicating that *L. plantarum* fermented SLBZS during this process. The results also showed the content of ginsenoside Rb1 in SLBZS increased significantly after fermentation. Ginsenoside Rb1 has been shown to have many benefits to the organism, including anti-oxidation, anti-stress, anti-inflammatory and so on (26, 41, 42). We suggested that *L. plantarum* fermentation of SLBZS could enhance its efficacy.

Next, we added 0.1% unfermented SLBZS and 0.05%, 0.1 and 0.2% fermented SLBZS to broiler feed, respectively. As far as we know, there is no report about SLBZS or fermented SLBZS on the growth performance of broilers, so we tested the growth performance of experimental broilers. Our results showed that medium and high doses of fermented SLBZS could significantly improve the ADFI of broilers and all three doses of fermented SLBZS could significantly improve the ADG of broilers in the second, third and whole stage, compared with the control group. Accordingly, the three doses of fermentation SLBZS in the second and the whole stage significantly reduced FCR, while the medium dose of fermentation SLBZS in the third stage significantly reduced FCR. ADFI, ADG and FCR are often used as indicators to evaluate growth performance (43, 44), so it can be concluded that fermentation of SLBZS could improve the growth performance of broilers. In addition, the supplementation of unfermented SLBZS tended to improve the growth performance of broilers, but there was no significant difference. During fermentation, the macromolecule or polymers forms of the active ingredient can be cut down to smaller molecule, which favors the transmembrane transport and improve adsorption of the active ingredients by the tissues (45). This may be the reason why the effect of SLBZS fermentation is better than that of unfermented SLBZS.

Broilers are vulnerable to pathogen invasion in early life, and pathogens preferentially attack lymphatic organs (46, 47). Relative lymphoid organ weight and immune factor content are often used to evaluate immune performance of broilers (48, 49). Next, relative lymphoid organ weight and serum IgA and IgG contents of broilers were measured. Higher relative lymphoid organ weight is considered to have greater immune performance (50, 51). In our experiment, in the second and third stages, medium dose and high dose of fermentation SLBZS could improve spleen, thymus and bursa of fabricius index. We observed that all three doses increased thymus index in the first stage, but had no significant effect on spleen and bursa of fabricius index. Both ginsenoside Rb1 and its intestinal metabolite Rh1 have been shown to regulate the balance of immune cells to inhibit infectious responses (52, 53). In the first stage, both unfermented and fermented SLBZS significantly increased the IgA content of broilers. However, we observed no significant change in IgA content in the second and third stages. IgA is the first line of defense for intestinal mucosal immunity (54), its increase is good for gut health. During the whole experiment, IgG content did not change significantly, which might be due to the fact that IgG was mainly involved in secondary immune response (55). In general, SLBZS could be metabolized more ginsenoside Rb1 by gut microbial after fermentation, which stimulates lymphocyte proliferation and improves immune function.

To a certain extent, antioxidant capacity can reflect the immune function and anti-infection ability of the organism (56). SOD, MDA, GSH-PX and T-AOC are commonly used to evaluate the antioxidant capacity of broilers (57, 58). In our study, unfermented SLBZS did not significantly affect the antioxidant capacity of broilers. Medium and high doses of SLBZS significantly increase the content of SOD in serum. Medium dose of fermented SLBZS significantly increase T-AOC of broilers, and high dose of fermented SLBZS significantly reduce MDA content. Ginsenosides Rb1 and Rh1 could prevent liver injury induced by APAP in mice and improve their antioxidant capacity (59). Ginsenosides Rg2 and Rh1 also enhance liver antioxidant capacity through Nrf2 pathway (60). In addition, *Lactobacillus* and its metabolite 5-methoxy-indoleacetic acid also enhance the antioxidant capacity of liver through Nrf2 (61). Therefore, we speculated that the release of more active components in fermented SLBZS and the presence of *Lactobacillus* could improve the antioxidant capacity of broilers.

The absorption function of small intestine is closely related to the growth performance of broilers (36), so we measured the intestinal morphology of ileum of broilers. We observed that medium and high doses of fermented SLBZS significantly increased VH:CD. Ji et al. found that SLBZS could significantly improve ileum villus density in diarrhea rats (62). This was also demonstrated by the significant increase in ileum villus height when adding unfermented SLBZS to the feed. A study has found that SLBZS could regulate the composition of gut microbiota in broilers (63). The increase of beneficial microbiota could produce more short-chain fatty acids (SCFA) (64) and promote the differentiation and proliferation of intestinal epithelial cells (65). Therefore, we suggest that fermentation of SLBZS can improve ileum VH/CD of broilers.

Tight junction proteins play an important role in maintaining the integrity of the intestinal barrier, helping to resist pathogen invasion of intestinal epithelial cells (31). Occludin-1 plays an important role in tight junction barrier function and signal transduction (66), and ZO-1 plays an important role in tight junction protein composition and maintenance of cell barrier permeability (67). Our study found that fermentation of SLBZS increased the relative expression of tight junction proteins. Fermentation of SLBZS produced increased saponins, which can better promote the proliferation of beneficial bacteria and produce more SCFA such as butyric acid. SCFA can increase AMPK activity in intestinal epithelial cells and accelerate the recombination of tight junction proteins (68). We hypothesized that SLBZS fermentation increased the expression of tight junction protein-related mRNA may cause by SCFA. Our previous studies have shown that SLBZS polysaccharide increase the abundance of SCFA-producing bacteria, increase the SCFA content in the intestine, and promote intestinal injury repair (22). According to our results, the abundance of *Coprococcus, Bifidobacterium, Bilophila, human* and *Blautia* in F-SLBZS were higher than those in Con and U-SLBZS. *Coprococcus* and *Bifidobacterium* are both butyric-producing bacteria (69). *Bilophila wexlerae* contains a high amount of starch (i.e., amylopectin) and produces carbohydrate metabolites (i.e., succinate, lactate, acetate) (70). As one of the metabolites of gut microbiota, SCFA is involved in the maintenance of intestinal barrier function and immune homeostasis (71). This may be one of the reasons for the better effect of fermented SLBZS.

In conclusion, this study optimized the fermentation conditions of *L. plantarum* for SLBZS, and fermented SLBZS could be a potential feed additive to promote the growth performance in broilers.

## Data availability statement

The sequences obtained by 16S rRNA sequencing were deposited in NCBI BioProject PRJNA906993, Short Read Archive (SRA SAMN31944550- SAMN31944561).

## Ethics statement

The animal study was reviewed and approved by Animal Ethics Committee of the South China Agricultural University.

## Author contributions

WL and SG designed the overall research experiments. WL, YM, YZ, TW, JH, SH, and HD performed the experiments. WL, YM, and HD analyzed the data. WL and YM wrote the manuscript. SG revised the manuscript. All authors contributed to the article and approved the submitted version.
