# Supplementary material for: Effects of Lactobacillus plantarum fermented Shenling Baizhu San on gut microbiota, antioxidant capacity, and intestinal barrier function of yellow-plumed broilers
Source: Front Vet Sci. 2023 Feb 22;10:1103023. doi: 10.3389/fvets.2023.1103023 (PMC9992544; doi:10.3389/fvets.2023.1103023)
Supplement: Supplementary file 1 [file Data_Sheet_1.docx]

**Effects of *Lactobacillus plantarum* Fermented Shenling Baizhu San on Growth Performance, Antioxidant Capacity, and Intestinal Barrier function of Yellow-plumed Broilers**

Weijie Lv ^1,2, †^, Yimu Ma ^1, †^, Yingwen Zhang ^1^, Tianze Wang ^1^, Jieyi Huang ^1^, Shiqi He ^1^, Hongliang Du ^1^, Shining Guo ^1, 2, *^

^1^ College of Veterinary Medicine, South China Agricultural University, Guangzhou, PRC.

^2^ Guangdong Technology Research center for Traditional Chinese Veterinary Medicine and Natural Medicine, Guangzhou, PRC.

^†^These authors contributed equally.

^*^ Correspondence: Shining Guo, E-mail address: shining@scau.edu.cn


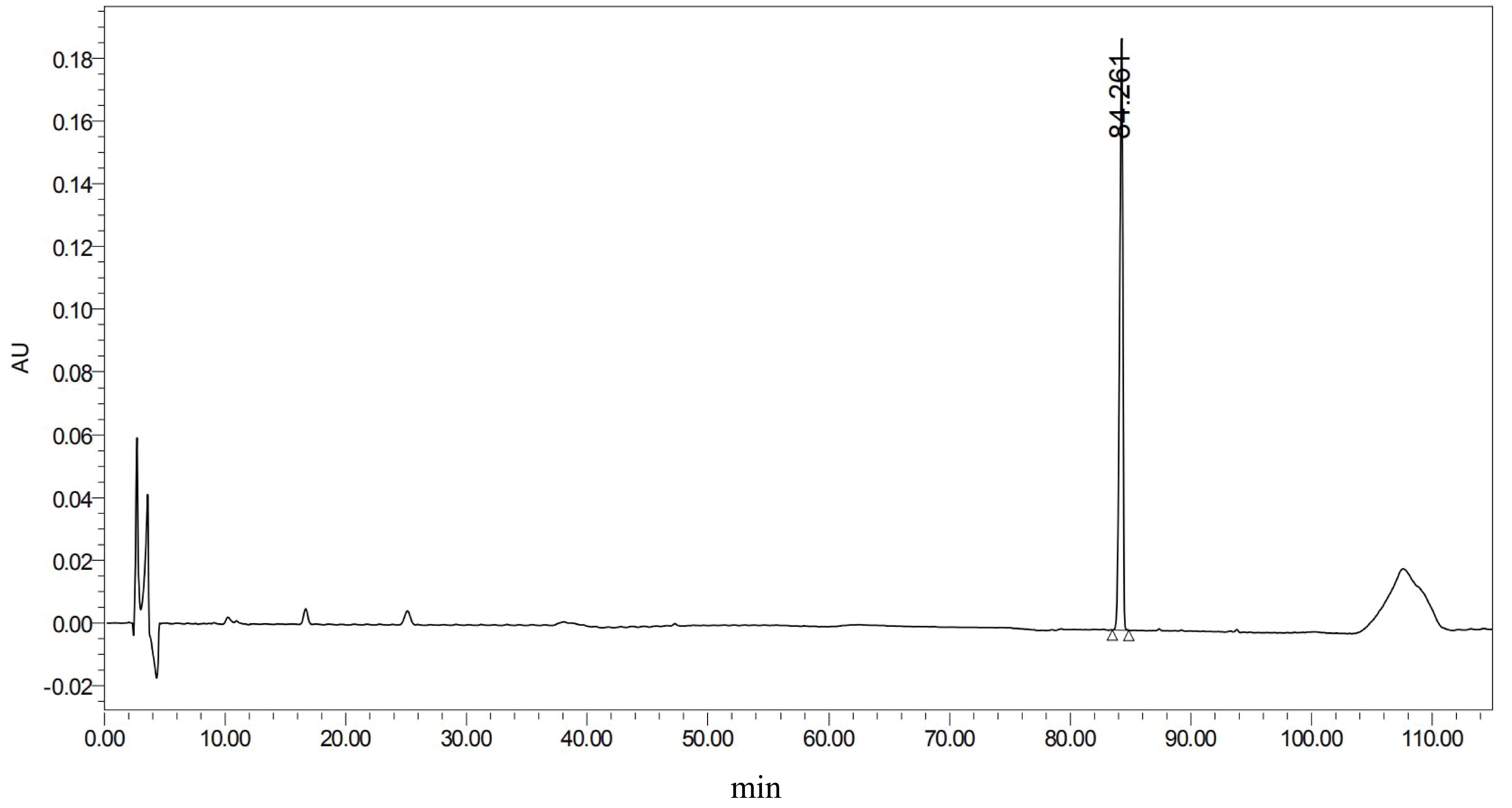


Figure S1: Chromatogram of ginsenoside Rb1 standard.


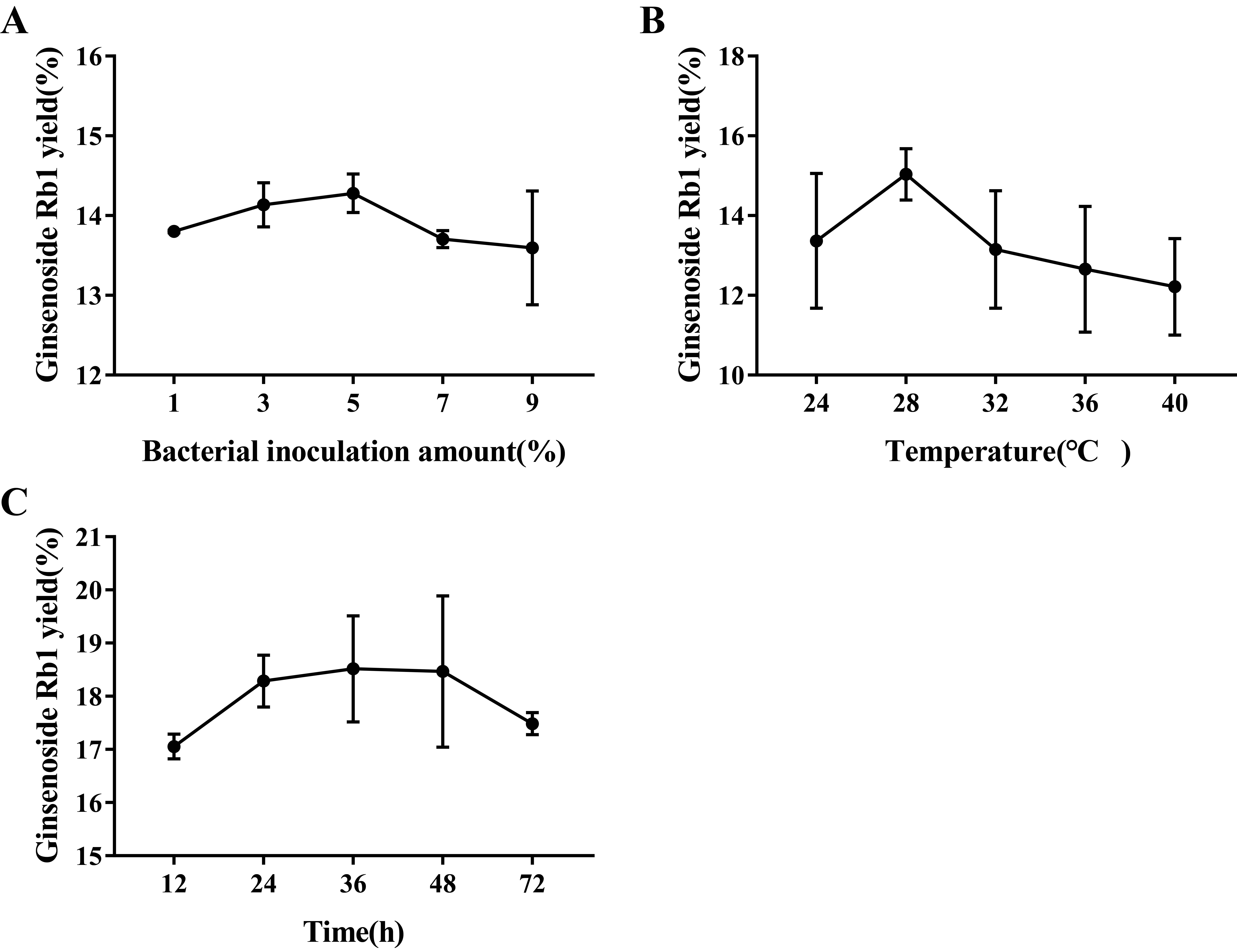


Figure S2: Effects of bacterial inoculation amount, fermentation temperature and fermentation time on ginsenoside Rb1 yield. (A) Effects of different bacterial inoculum amount on ginsenoside Rb1 yield. (B) Effects of different fermentation temperature on ginsenoside Rb1 yield. (C) Effects of different fermentation time on ginsenoside Rb1 yield.


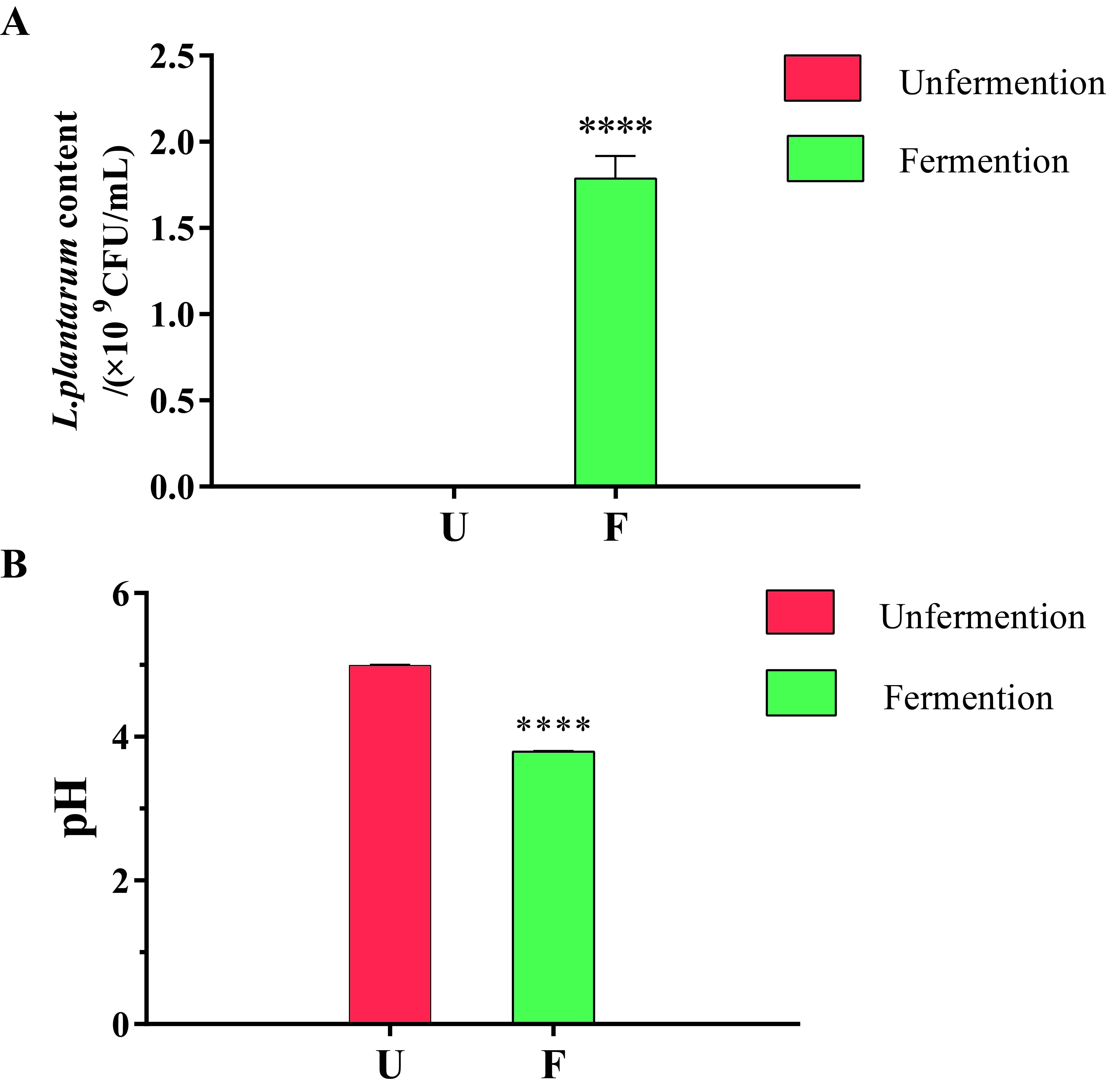


Figure S3: Changes of *L.plantarum* content and pH value before and after SLBZS fermentation. (A) The content of Lactobacillus plantarum in SLBZS before and after fermentation. (B) The pH of SLBZS before and after fermentation. Bars represent mean values ± SD (n = 3). **** indicate *p* < 0.0001 compared with the Unfermention.


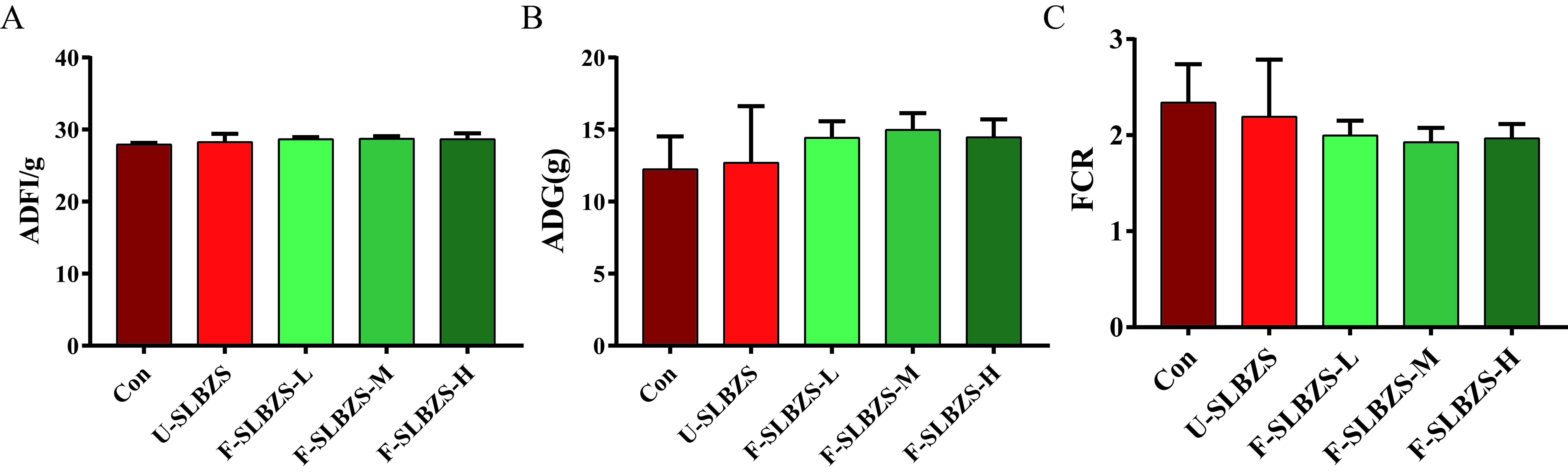


Figure S4: ADFI, ADG and FCR of broilers in the first stage. (A) ADFI of broilers in the first stage; (B) ADG of broilers in the first stage; (C) FCR of broilers in the first stage.


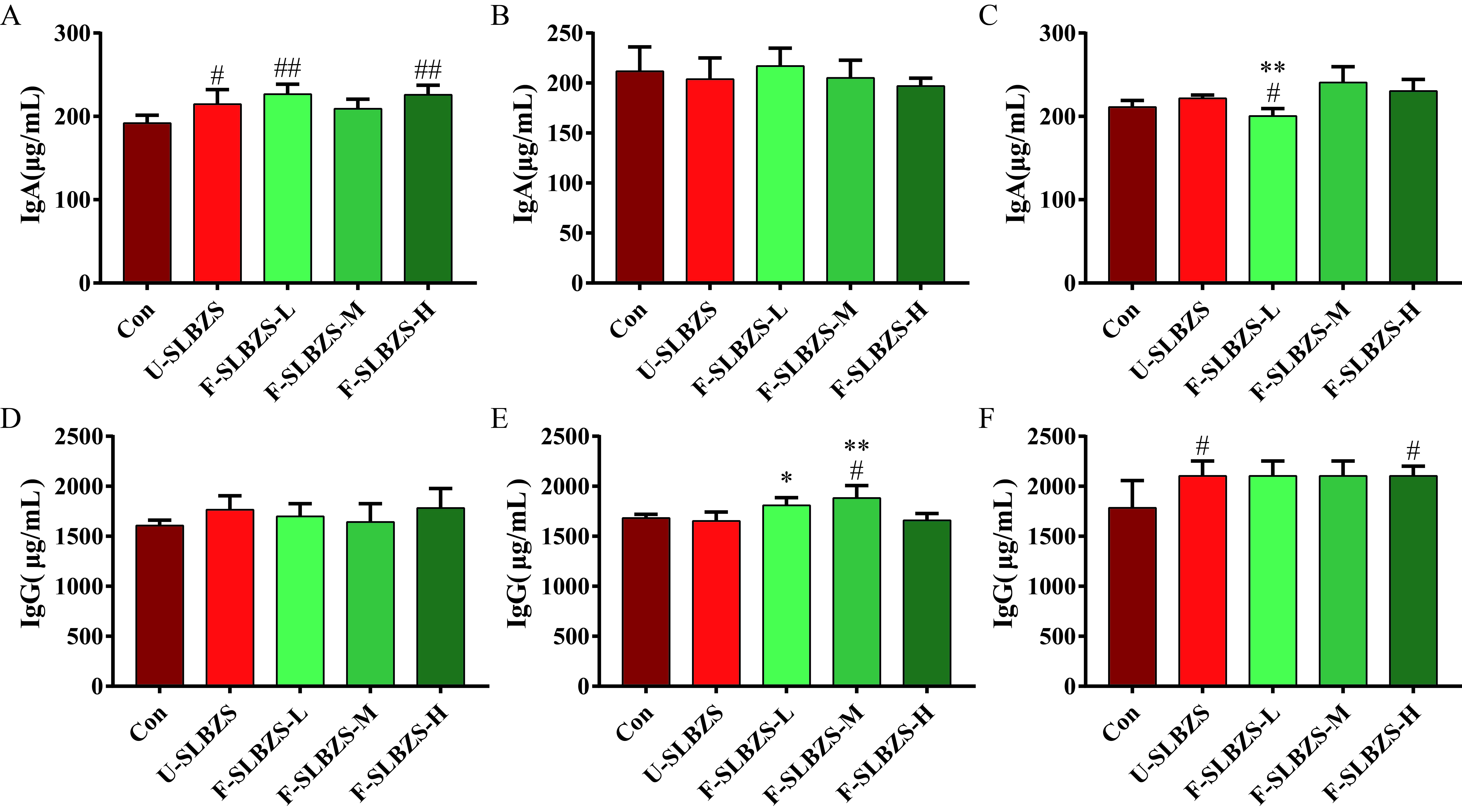


Figure S5: Effects of SLBZS fermentation on IgA and IgG contents in broilers. (A) The content of IgA in serum of broilers on day 14; (B) The content of IgA in serum of broilers on day 28; (C) The content of IgA in serum of broilers on day 42; (D) The content of IgG in serum of broilers on day 14; (E) The content of IgG in serum of broilers on day 28; (F) The content of IgG in serum of broilers on day 42. Bars represent mean values ± SD (n = 3–5). # and ## indicate *p* < 0.05 and *p* < 0.01 compared with the Con group; * and ** indicate *p* < 0.05 and *p* < 0.01 compared with the U-SLBZS group.

Table S1: The gradient elution procedure

| Time/min | Mobile phase A% | Mobile phase B% |
| --- | --- | --- |
| 0 | 81 | 19 |
| 35 | 81 | 19 |
| 55 | 71 | 29 |
| 70 | 71 | 29 |
| 100 | 60 | 40 |
| 105 | 81 | 19 |
| 115 | 81 | 19 |

Table S2: Single factor levels

| Factors | Levels | | | | |  |
| --- | --- | --- | --- | --- | --- | --- |
| Bacterial inoculum | 1% | 3% | 5% | 7% | 9% | |
| Fermentation temperature | 24℃ | 28℃ | 32℃ | 36℃ | 40℃ | |
| Fermentation time | 12 h | 24 h | 36 h | 48 h | 72 h | |

Table S3: Composition and nutrient level of feed.

| Raw material composition | Content/% | | Main nutritional indicators | Content/% | |
| --- | --- | --- | --- | --- | --- |
|  | 1-28 d | 29-42 d |  | 1-28 d | 29-42 d |
| Corn | 46 | 27.5 | Metabolic energy (Kcal/Kg) | 2900 | 2900 |
| Soybean meal 46% | 24.5 | 15 | Crude protein/% | 19.5 | 17.5 |
| Barley | 15 | 25 | Calcium/% | 1.05 | 0.95 |
| Rice bran | 0 | 5 | The effective phosphorus/% | 0.45 | 0.42 |
| Bran | 5 | 12 | Lysine/% | 1.02 | 0.9 |
| Corn gluten meal | 4 | 4 | Methionine/% | 0.45 | 0.42 |
| Oil and grease | 1.5 | 3.5 |  |  |  |
| Distillers dried grains with solubles | 0 | 4 |  |  |  |
| Premix | 4 | 4 |  |  |  |

Table S4: The levels of each factor in the orthogonal test

| Levels | A: Bacterial inoculation amount | B: Fermentation temperature | C: Fermentation time |
| --- | --- | --- | --- |
| 1 | 3% | 24 ℃ | 24 h |
| 2 | 5% | 28 ℃ | 36 h |
| 3 | 7% | 32 ℃ | 48 h |

Table S5: Sequences of the forward (F: 5’ → 3’) and reverse (R: 5’ → 3’) primers designed for the GAPDH, Claudin-1, Occludin, ZO-1 genes.

| Gene | Full Name | Primer sequence |
| --- | --- | --- |
| GAPDH | glyceraldehyde-3-phosphate dehydrogenase | F: GGCACGCCATCACTATC |
|  |  | R: CCTGCATCTGCCCATTT |
| Claudin-1 | / | F: TGGCCACGTCATGGTATGG |
|  |  | R: AACGGGTGTGAAAGGGTCATAG |
| Occludin | / | F: ACGGCAGCACCTACCTCAA |
|  |  | R: GGGCGAAGAAGCAGATGAG |
| ZO-1 | Zonula Occludens-1 | F: CCGCAGTCGTTCACGATCT |
|  |  | R: GGAGAATGTCTGGAATGGTCTGA |
